# Lessons learnt from implementing community engagement interventions in mobile hard-to-reach (HTR) projects in Nigeria, 2014–2015

**DOI:** 10.1186/s12889-018-6193-z

**Published:** 2018-12-13

**Authors:** Kulchumi Isa Hammanyero, Samuel Bawa, Fiona Braka, Bassey Enya Bassey, Akinola Fatiregun, Charity Warigon, Yared G. Yehualashet, Sisay Gashu Tegene, Richard Banda, Charles Korir, Tesfaye Bedada Erbeto, Martin Chukwuji, Pascal Mkanda, Usman Saidu Adamu, Peter Nsubuga

**Affiliations:** 1 World Health Organization, Country Representative Office, Abuja, Nigeria; 2 World Health Organization, Regional Office for Africa, Brazzaville, Congo; 3 National Primary Health Care Development Agency (NPHCDA), Abuja, Nigeria; 4 Global Public Health Solutions, Atlanta, GA USA

**Keywords:** CE, Hard to reach, Mobile health team, Mobile immunization services

## Abstract

**Background:**

The year 2014 was a turning point for polio eradication in Nigeria. Confronted with the challenges of increased numbers of polio cases detected in rural, hard-to-reach (HTR), and security-compromised areas of northern Nigeria, the Nigeria polio program introduced the HTR project in four northern states to provide immunization and maternal and child health services in these communities. The project was set up to improve population immunity, increase oral polio vaccine (OPV) and other immunization uptake, and to support Nigeria’s efforts to interrupt polio transmission by 2015. Furthermore, the project also aimed to create demand for these services which were often unavailable in the HTR areas. To this end, the program developed a community engagement (CE) strategy to create awareness about the services being provided by the project. The term HTR is operationally defined as geographically difficult terrain, with any of the following criteria: having inter-ward/inter-Local Government Area/interstate borders, scattered households, nomadic population, or waterlogged/riverine area, with no easy to access to healthcare facilities and insecurity.

**Methods:**

We evaluated the outcome of CE activities in Kano, Bauchi, Borno, and Yobe states to examine the methods and processes that helped to increase OPV and third pentavalent (penta3) immunization coverage in areas of implementation. We also assessed the number of community engagers who mobilized caregivers to vaccination posts and the service satisfaction for the performance of the community engagers.

**Results:**

Penta3 coverage was at 22% in the first quarter of project implementation and increased to 62% by the fourth quarter of project implementation. OPV coverage also increased from 54% in the first quarter to 76% in the last quarter of the 1-year project implementation.

**Conclusions:**

The systematic implementation of a CE strategy that focused on planning and working with community structures and community engagers in immunization activities assisted in increasing OPV and penta3 immunization coverage.

## Background

With the Alma Ata Declaration in 1978, community engagement (CE) (or participation, as interchangeably used) has become a cardinal principle in primary healthcare (PHC) in Nigeria. The primary care agencies at the national, state, or local levels have the mandate to deliver PHC services in a manner that is community driven, community operated, and community owned [1]. CE is also the fourth component of the reaching every ward (REW) strategy, which encourages the participation of community members and groups in the planning, implementation, and monitoring of routine immunization programs [2]. Furthermore, the Global Vaccine Action Plan states that CE presupposes that “individuals and communities understand the value of vaccines and demand immunization as both their right and responsibility” [3, 4].

A number of studies on community mobilization for maternal and newborn child health have demonstrated how CE and participation are important for the delivery of community-based child survival interventions for the mother, newborn, and children [5–8]. However, if CE is not performed systematically in primary-care programs or is simply seen as a useful but nonessential companion to the delivery of treatments and preventive health education, it is bound to fail in achieving its objectives [7, 9].

Additionally, poor CE planning combined low investment have been found to be responsible for poor health outcomes of the beneficiaries at both individual and communal particularly for marginalized communities such as the hard-to-reach (HTR) areas [10].

The Government of Nigeria, with support from the World Health Organization (WHO) and funding from the Bill and Melinda Gates Foundation (BMGF), implemented the integrated maternal, newborn, and child health (MNCH) project in the HTR areas. In this context, the HTR areas are operationally defined as geographically difficult terrain, with any of the following criteria: having inter-ward, inter-Local Government Area (LGA), or interstate borders, scattered households, a nomadic population, or a waterlogged, riverine area, with no easy to access to healthcare facilities and insecurity.

CE was implemented as a key component of the HTR project. The engagement was an ongoing interactive process that engaged beneficiary HTR communities in the planning and implementation of maternal, neonatal, and child health services in a manner that addressed the demand of side factors using existing community structures to improve coverage.

In this paper, we describe the processes and methods for engaging the communities, and assess the performance of CE structures and their impact on improving immunization coverage in Bauchi, Borno, Kano, and Yobe states of northern Nigeria.

## Methods

We conducted a descriptive, retrospective study that reviewed HTR data from June 2014 to June 2015 to determine the contribution of CE interventions to increasing oral polio vaccine (OPV) and the third dose of the pentavalent vaccine (penta3) coverage. Pentavalent vaccine is a five-in-one vaccine used to protect children against diphtheria, tetanus, pertussis, hepatitis B, and *Haemophilus influenza* type B.

The study locations for this study were the states of Kano, Borno, Yobe, and Bauchi. The aim of the study is to showcase the methods and processes used to improve immunization results in a time-bound project with a view to replicating it in routine immunization.

### Planning

We conducted a baseline survey in the four selected states using a focus group discussion (FGD) and key informant interviews (KIIs) to identify the needs of the community and the channels for reaching them with information regarding immunization services as part of planning with the community. The findings from FGDs and 174 KIIs conducted revealed that town announcers and community mobilizers were the best channels for mobilizing the communities for immunization.

Based on findings from the baseline study, we developed a module to train HTR personnel, town announcers, and mobilizers to ensure that all personnel involved in the implementation of the project were familiar with the concept and importance of CE.

The mobile HTR team leaders enumerated all households in selected settlements during a micro-plan process to ensure early planning and community involvement and participation in the project. During this process, mobile health teams (MHT) convened meetings with community leaders to identify qualified town announcers and community mobilizers to operate in each assigned settlement. We used such fora to identify days, dates, and locations of immunization posts and identified town announcers, community mobilizers, and other community structures such as village development committees (VDCs). This process was done in all the settlements selected for the HTR.

Before the full implementation of the project, we trained the HTR personnel using the CE module on their roles and responsibilities in CE, the benefits, and the importance of ensuring community involvement in the implementation of the project.

Before every session, each MHT leader met with community leaders, community-based organizations, town announcers, community mobilizers, women, youths, and VDC members to sensitize them on the importance and benefits of immunization. The need to track defaulters and the referral of newborns to vaccination posts was also discussed.

Although VDCs were not available in all the 2311 settlements targeted for the project, where they were available the MHT encouraged them to have meetings to discuss issues of vaccine dropout, mobilization of the communities, and tracking of newborns.

### Implementation

Before the commencement of each session, trained town announcers and community mobilizers went around their communities to mobilize caregivers to vaccination posts. The town announcers and mobilizers gave information about the dates, time, locations, and antigens provided at vaccination posts. As the town announcers went around the communities, the community mobilizers visited each house in the community to converse with caregivers about the HTR services and responded to the concerns of caregivers. Based on the orientation that was given to them, community mobilizers referred newborns and pregnant women to vaccination posts.

Traditional leaders who served as the link between MHT and communities mobilized communities at mosques, churches, and public gatherings by informing their people about the dates and locations of immunization services.

Where they existed, VDC members mobilized communities to mobile outreach sessions. The VDC helped to create awareness, stimulate demand, and convince noncompliant communities to accept immunization. The VDC members also tracked defaulters based on the data provided by the MHT.

### Post-implementation evaluation

Data were collected weekly using supervisory checklists. All supervisors, including the team leader for the mobile outreach, transmitted data in real time to enable quick analysis and action. Hardcopies of the checklists were also collected and analyzed.

Indicators such as the number of community mobilizers, town announcers involved in mobile sessions, the presence and activities of the community-based organizations, VDCs, and traditional leaders in mobilization were collated and analyzed to inform the program action. We conducted a mid-term review (MTR) in November 2015 to assess the progress of the HTR project. We administered structured questionnaires at the health facility and community levels to assess, among other issues, the level of CE and the utilization of services. We administered questionnaires to 2311 households and 231 traditional leaders.

## Results

In the 2311 settlements selected to implement the HTR project (Table 1), a total of 4622 town announcers and community mobilizers were involved in mobilizing and creating demand for mobile outreach services. A total of 1170 community-based organizations (CBOs) and 431 VDCs were involved in the mobilization of the settlements during the period 2014 to 2015. The number of community leaders that mobilized caregivers to vaccination posts was 2975.

**Table 1:** State distribution of community-based structures June 2014 to June 2015

State	Settlements targeted	No. of community mobilizers engaged	No. of town announcers engaged	No. of CBA/CBOs	No. of community leaders involved	No. of VDCs involved
Bauchi	763	763	763	993	994	330
Borno	620	620	620	30	940	39
Kano	406	410	410	139	511	59
Yobe	522	518	518	8	530	3
Total	2311	2311	2311	1170	2975	431

*CBA* community based association, *CBO* community-based organization, *VDC* village development committee

In the opinion of the caregivers, 45% responded that the community mobilizer was the person that mobilized them to the vaccination posts (Table 2), followed by the town announcer (40%) and community leaders (30%). The result, however, varied across the states since community mobilizers in Bauchi, Yobe, and Borno scored 63, 54, and 38%, respectively, while in Kano town announcers (64%) were the main mobilizers.

**Table 2:** Outcome results from the activities of community mobilizers, town announcers, and other community structures from June 2014 to June 2016 in the hard-to-reach (HTR) areas of Bauchi, Kano, Yobe, and Borno

State	Satisfied with the services provided?		Mobile outreach attendance	Person who mobilizes household			Knowledge about the mobile HTR team outreach session conducted?		
	Mobilizer ever visited household for health activities?	Satisfied	Attendance at mobile health team outreach sessions	Town announcer	Community leader	Community mobilizer	Town announcer	Community leader	Community mobilizer
Bauchi	92% (635)	99% (682)	96% (659)	61% (385)	59% (374)	63% (401)	62% (406)	72% (475)	50% (331)
Borno	66% (399)	97% (590)	82% (498)	14% (56)	17% (69)	38% (152)	17% (85)	58% (288)	12% (58)
Kano	83% (463)	97% (545)	98% (548)	64% (298)	21% (97)	26% (122)	90% (492)	29% (159)	20% (111)
Yobe	53% (263)	81% (398)	79% (391)	22% (59)	21% (54)	54% (143)	34% (134)	43% (169)	36% (141)
Total	74% (1760)	94% (2215)	89% (2096)	40% (798)	30% (594)	45% (818)	51% (1117)	51% (1091)	30% (641)

Values are shown as % (*n*)

For knowledge about the services of the mobile outreach team, overall 51% of caregivers identified community leaders and town announcers as the main sources of information about mobile outreach services. However, in Bauchi 72% identified community leaders, with values of 90% for town announcers in Kano, 58% for community leaders in Borno, and 43% for community leaders in Yobe.

Customer service satisfaction with the work of community mobilizers was > 80% in all four states; with a high of 99% in Bauchi, 97% each in Borno and Kano, and the lowest in Yobe at 81%. Caregivers interviewed reported that community mobilizers (74%) had visited their homes to mobilize them on health matters. Bauchi reported more community mobilizers (92%) visited their homes, followed by Kano (83%) and Borno (66%), with the lowest reports for Yobe (at 53%).

With the introduction of the project in the first quarter, OPV coverage for children below 1 year of age was 44%; however, this figure reduced in the second quarter but steadily increased to 54 and 76% by the fourth quarter. There are, however, variations in the number of children vaccinated against OPV by each state. While some states such as Kano and Yobe started the year with low coverage (17 and 18%, respectively), their coverage increased to 40 and 167% respectively by last quarter. Bauchi and Borno, on the other hand, started at 36 and 80%, respectively. Over the next three quarters, Bauchi coverage increased steadily to 48, 74, and 77% by the fourth quarter. However, Borno recorded a decrease from 80 to 61% by the fourth quarter.

Penta3 coverage in the first quarter for all states was 22% but, by the last quarter of the first year of the project (2015), it rose to 62%. A similar trend in coverage was also observed across the four states, with Bauchi having the highest (28%) while Yobe recorded the lowest (6%) in the first quarter. However, by the second quarter the coverage in Yobe rose from 6 to 42%, while Bauchi recorded 41, 55, and 54% in the second, third, and fourth quarters, respectively. Similar to OPV coverage, Kano recorded (Table 3).

**Table 3:** Distribution of OPV and Penta coverage in the selected HTR settlements of Bauchi, Kano, Yobe and Borno June 2014 – June 2015

State	Jun-Sep 14	Oct-Dec 15	Jan-Mar 15	Apr - Jun 15	Jun-Sep 14		Oct-Dec 15			Jan-Mar 15			Apr-Jun 15			
	OPV < 1	OPV < 1	OPV < 1	OPV < 1	Penta1	Penta2	Penta3	Penta1	Penta2	Penat3	Penta1	Penta2	Pent3	Penta1	Penta2	Penta3
Bauchi	3776 (36%)	5002 (48%)	7786 (74%)	8076 (77%)	7388 (70%)	3341 (32%)	2906 (28%)	6467 (62%)	5824 (55%)	4273 (41%)	7069 (67%)	5498 (52%)	5753 (55%)	6750 (64%)	5081 (48%)	5653 (54%)
Borno	9623 (80%)	5306 (44%)	5568 (46%)	7359 (61%)	10,524 (87%)	5572 (46%)	4278 (36%)	5691 (47%)	5030 (42%)	3748 (31%)	5460 (45%)	4059 (34%)	2972 (25%)	7635 (64%)	6113 (51%)	5707 (47%)
Kano	875 (17%)	916 (18%)	2013 (39%)	8590 (167%)	3430 (67%)	790 (15%)	459 (9%)	2122 (41%)	1863 (38%)	817 (16%)	1588 (31%)	1368 (27%)	2013 (39%)	6279 (122%)	5110 (99%)	8568 (166%)
Yobe	1531 (18%)	4022 (47%)	4382 (51%)	3397 (40%)	7689 (90%)	2573 (30%)	519 (6%)	3070 (36%)	4374 (51%)	3626 (42%)	3440 (40%)	2693 (31%)	3971 (46%)	3792 (44%)	2692 (31%)	2641 (31%)
Total	15,805 (44%)	15,246 (42%)	19,749 (54%)	27,422 (76%)	29,031 (80%)	12,276 (34%)	8162 (22%)	17,350 (48%)	17,191 (47%)	12,464 (34%)	17,557 (48%)	13,618 (38%)	14,709 (41%)	24,456 (67%)	18,996 (52%)	22,569 (62%)

## Discussion

We found that penta3 coverage increased from 22 to 62% by the fourth quarter of the project cycle due to the systematic engagement of existing community structures in the HTR areas. We also observed that OPV coverage of children less than 1 year of age in the HTR settlements increased from 44% in the first quarter to 76% in the last quarter of the 1-year project implementation. There was, however, a fall in the results in the second and third quarters of the project period, with a significantly high drop noticeable in Borno from 80 to 61% thereby affecting the overall performance of the four states. We also found that most of the sampled households had visits by the community mobilizers to sensitize caregivers about the services provided by the HTR teams.

Regarding the use of community structures to create awareness and demand for mobile outreach services, Bauchi recorded the highest figures regarding the use of town announcers, community mobilizers, and traditional rulers to mobilize hard-to-reach communities to vaccination. It is not surprising that this state had the highest household visits (92%) during the period under review since it engaged the highest number of town criers compared with all the other states. Some studies have reported that the use of community structures is the most cost-effective and cheapest means of passing information to caregivers. For example, a community study in southern Nigeria found that town announcers (often referred as town criers) can be as effective as community extension workers if properly supported [11].

With regards to the presence and participation of VDCs in mobilizing communities for mobile outreach services, where these were available states took advantage of their existence to plan and implement mobile outreach sessions. A study from a project in Cambodia found that donor-funded projects need to work with “existing community-based organizations or agencies” to mobilize communities, particularly if the project has a short time frame [6]. One of the reasons for working with VDCs in the HTR areas is to ensure community ownership and continuity of CE activities even after the project has ended.

Although we identified some indicators at the beginning of the project that we used to assess the use of the community structures, we did not assess the impact or the outcome of these structures until the MTR was conducted in 2015. From the results of the MTR, caregivers have expressed satisfaction with the work of the community mobilizers recruited to mobilize their communities. Satisfaction for the work of community mobilizers was highest in Bauchi (63%) and lowest in Kano (26%). It is not surprising to observe that Bauchi recorded more beneficiary satisfaction since the state has the highest number of settlements and each settlement has a community mobilizer, town announcer, and traditional leader. Having a community mobilizer who is respected and resident in the community helped in the passage of the right information for caregivers to act by visiting vaccination posts. Perhaps the most important point about our CE approach was not just about raising awareness about the services of the HTR project, but also persuading community members to take action by visiting vaccination posts.

We found out that CE interventions implemented in the HTR settlements were instrumental to the steady increase in immunization coverage in these areas. Implementation of community activities during the period under review showed improved routine immunization outcomes with an increase from 22% penta3 coverage in the first quarter to 62% by the fourth quarter within the first year of project implementation. When compared with the service satisfaction of clients about the HTR and the CE activities, the data analysis showed that Bauchi had met all three indicators. This is indicative of the fact that CE, when implemented alongside other interventions, has the potential to improve coverage, particularly for marginalized communities such as the HTR population [5, 10, 12–15].

One of the ways of monitoring immunization coverage is through the monitoring of penta3 vaccine administration to children less than 1 year old. As a proxy for routine immunization coverage, penta3 coverage increased dramatically in the HTR states from the first quarter, when penta3 coverage was 22, to 62% by the fourth quarter. These results varied across states, with Bauchi recording a realistic increment from 28 to 54% by the fourth quarter, followed by Borno which increased from 36% in the first quarter to 47% by the fourth quarter. The lowest in the first quarter was Yobe with 6%, but this steadily increased to 31% and was thus the state with the highest increase. While there was a steady increase in penta3 in all states, except for Kano where the increase was from 9 to 166%. This may be the result of data quality issues including denominator values. A tool developed by the WHO data quality audit (DQA) which assesses the quality and efficiency of immunization performance could have been used to identify the problems [13].

Although there is no direct link with the coverage data, the evidence presented elsewhere shows that when CE interventions are planned and implemented properly it “can lead to improved health and health behaviours among disadvantaged populations” [5]. Studies have shown that when community-based organizations are involved in the demand for creative activities, they tend to strengthen the communication and accountability lines between the health workers and the community. Engagement with community-based organizations such as VDCs, community leaders, community mobilizers, town announcers, and other community structures are instrumental in enhancing demand for immunization services [15, 16].

Our report is subject to at least three major limitations to its generalization beyond our context. First, while we are aware that CE activities alone are not enough to increase coverage, we recognize that other extraneous factors such as the provision of incentives in the form of child survival interventions including treatment of minor ailments might have influenced the outcome of the results. A study of knowledge, attitude, and practice conducted in Borno revealed that incentives are a major influence in the decision of mothers to vaccinate their children [17]. In addition, improved service delivery and exhibition of interpersonal communication skills by the mobile health teams might have contributed to the results we saw in HTR immunization coverage.

Secondly, we are aware of the fact that routine immunization coverage data are not complete if they are not accompanied by an analysis of the dropout rate. Dropout determines the number of children that have started immunization but could not complete it for various reasons.

Thirdly, the HTR project is an ongoing project, but we restricted our study to the period 2014 to 2015, the initial lifespan of the project. Other interventions and activities after June 2015 might have influenced the outcome of the findings of the MTR.

For researchers to be able to demonstrate the direct impact of CE initiatives accurately, it is important to undertake further study to measure the direct impact of the CE activities on coverage and change in people’s behavior.

With the current government plan to implement a “PHC under one roof” (PHCUR) policy, the lessons from this study will help to shed light on how communities in the HTR and other difficult areas can be included in planning for mobile outreach services. Similarly, other time-bound projects could learn from the methods of this project for ways they can harvest CE results to increase immunization coverage within a stipulated time.

## Conclusion

This paper describes the approach and processes that we adopted to engage communities of HTR areas. The step-by-step process approach not only yielded results regarding client service satisfaction, but also showed its effects on coverage of the two most important interventions of the project: OPV and penta. The lessons from the implementation of CE interventions in the areas that are HTR make a case for practitioners or groups to adopt the methods demonstrated in this study, particularly if the project is time bound.

## Abbreviations

BMGF: Bill and Melinda Gates Foundation
CBO: Community-based organization
CE: Community engagement
DQA: Data quality audit
FGD: Focus group discussion
HTR: Hard-to-reach
KII: Key informant interview
LGA: Local Government Area
MHT: Mobile health teams
MNCH: Maternal, newborn, and child health
MTR: Mid-term review
OPV: Oral polio vaccine
Penta: Pentavalent vaccine
PHC: Primary healthcare
REW: Reaching every ward
VDC: Village development committee
WHO: World Health Organization
